# Crystalline Sponge Method by Three-Dimensional Electron Diffraction

**DOI:** 10.3389/fmolb.2021.821927

**Published:** 2022-02-07

**Authors:** Pohua Chen, Yang Liu, Chaochao Zhang, Fei Huang, Leifeng Liu, Junliang Sun

**Affiliations:** ^1^ College of Chemistry and Molecular Engineering, Beijing National Laboratory for Molecular Sciences, Peking University, Beijing, China; ^2^ ReadCrystal Technology Co., Jiangsu, China

**Keywords:** crystalline sponge, electron diffraction, host-guest interaction, porous materials, structure solution

## Abstract

The crystalline sponge method has shown to be a novel strategy for the structure determination of noncrystalline, oily, or trace amount of a compound. A target compound was absorbed and oriented orderly in the pregrown porous crystal for x-ray diffraction analysis. However, the diffusion in the micron-sized crystals is rather difficult. Lots of trial-and-error experiments are needed to optimize the guest-soaking process and to improve data quality. Nanocrystals are better in diffusion, yet it could not conduct a single crystal x-ray diffraction (SCXRD) analysis. Three-dimensional electron diffraction (3D-ED) is a powerful diffraction tool for the structure determination of small crystals. In this work, we successfully carried out the crystalline sponge method by 3D-ED technique using {(ZnI_2_)_3_-[2,4,6-tris(4-pyridyl)-1,3,5-triazine]_2_·x(guest)}_n_ (1-Guest) porous complex nanocrystals. On account of the better diffuse ability of nanocrystals, the time needed for solvent exchange and guest soaking protocols are shortened 50-fold faster versus the original protocol. The crystal structure of the crystalline sponge incorporated with three different guests was fully resolved using a merged dataset. The structure model was identical to previously reported ones using x-ray, showing that the accuracy of the 3D-ED was comparable with SCXRD. The refinement results can also give the precise occupancy of the guest molecule soaked in the porous crystal. This work not only provides a new data collection strategy for crystalline sponge method but also demonstrates the potential of 3D-ED techniques to study host-guest interaction by solving the fine structure of porous material.

## Introduction

A molecular structure of a substance determines its intrinsic properties, such as reactivity, stereochemistry selectivity, etc. Chemists have developed nuclear magnetic resonance spectroscopy (NMR) and mass spectroscopy (MS) to provide essential structural information, but there are still chances to assign the structure incorrectly. Single-crystal x-ray diffraction (SCXRD) is the standard method that provides a direct three-dimensional structure at the atomic level. However, the major limitation of SCXRD is that it requires the target compound to crystallize into a single crystal with sufficient quality and size (>5 μm × 5 μm × 5 μm).

To address the challenge of structure determination on noncrystalline samples, Fujita and coworkers reported the “crystalline sponge” (CS) method (Inokuma et al., 2013; Hoshino et al., 2016). The CSs are porous crystalline materials that can absorb molecules in the solution and enable them to orient orderly within the pore. By soaking the molecule of interest into the pregrown CS single crystal, SCXRD can be performed on the host–guest complex, and therefore, the structure of the molecule of interest and the host can be elucidate simultaneously. The most commonly used CS is [(ZnI_2_)_3_-(tpt)_2_·x(Guest)]_n_ porous complex [noted as *1-Guest* **,** = 2,4,6-tris(4-pyridyl)-1,3,5-triazine]. The CS method has successfully determined the structure of natural products (Urban et al., 2016), metabolites (Zigon et al., 2015), and pharmaceutical compounds (Taniguchi et al., 2020). This method can also determine the absolute configuration of the organic compound of interest (Sairenji et al., 2017; Yan et al., 2017). However, the CS method is facing two major drawbacks. The yield of the porous host single crystal suitable for the CS method is quite low. More importantly, the critical guest-soaking step still needs trial-and-error experiments to optimize (Hoshino et al., 2016). Unoptimized soaking conditions, such as soaking time, temperature, and evaporation rate, may lead to crystal cracking and lowering guest diffusion, thus decreasing the chance of success structure determination on the molecule of interest.

Three-dimensional electron diffraction (3D-ED) is an emerging technique that collects single-crystal diffraction data from a nanocrystal (Gemmi et al., 2019; Gemmi and Lanza, 2019). It has demonstrated its powerful ability on determining complex structures including zeolites (Su et al., 2014), metal–organic frameworks (MOFs) (Huang et al., 2021), covalent organic frameworks (COFs) (Huang et al., 2021), and proteins (Nannenga and Gonen, 2019; Nguyen and Gonen, 2020). Combining a continuous rotation collection protocol, a noise-free direct electron detector, and a cryogenic sample holder, a single 3D-ED dataset could be recorded in 3 min with minimized beam damage (Nannenga et al., 2014; Nannenga and Gonen, 2019). Although many unknown structures were studied by 3D-ED, the host–guest interaction studies by 3D-ED were less reported. Owing to the vacuum environment and beam damage, the guest molecules more easily escaped from the host crystal. Until now, only few studies reported the identification of guests in the porous materials (Wang et al., 2018), or ligand binding in proteins (Clark et al., 2021).

In this work, we demonstrate the crystalline sponge method using the 3D-ED technique with nanosized CS crystal. Using a merged dataset with high completeness and resolution, the position of the framework and the guest molecule can be resolved *ab initio* and have comparable accuracy with a traditional x-ray diffraction method.

## Materials and Methods

### Materials

2,4,6-Tris(4-pyridyl)-1,3,5-triazine was purchased from the Energy Chemical Co. ZnI_2_ and guaiazulene were purchased from the Shanghai Aladdin Biochemical Technology Co., Ltd. All solvents used were purchased from Energy Chemical Co.

### Host Synthesis and Guest Inclusion Procedures

The [(ZnI_2_)_3_-(tpt)_2_·x(Guest)]_n_ porous complex [tpt = 2,4,6-tris(4-pyridyl)-1,3,5-triazine] (donated as *1-Guest*) host was synthesized following the protocol of Fujita (Hoshino et al., 2016). The crystalline sponge was prepared by layering a methanol solution of ZnI_2_ (14.4 mg in 1.5 ml) on a nitrobenzene solution of (9.5 mg in 6 ml). The solution was left for 7 days for crystallization. The powder-like *1-Nitrobezene* at the bottom of the bottle was transferred to a new glass vial. It was then washed 2–3 times cyclohexane using the same volume of the synthesis liquid. The powder-like crystals were heated in cyclohexane at 50°C for 2 h and stored in the same liquid after it cooled to room temperature. The solvent exchange process was monitored by IR spectroscopy (Supplementary Figure S1). To include guest, about 1 mg of *1-Cyclohexane* crystal was added into a capped glass vial. The excess solvent was removed carefully. Then 1 ml of guaiazulene cyclohexane solution (concentration ∼1 mg/ml) was added in the vial. The pierced capped vial was heated at 50°C for 12 h and transferred to 4°C for storage. This sample was noted as *1-Guaiazulene*.

### 3D-ED Data Collection and Processing

The crystal suspension was drop-casted onto a copper grid (R1.2/1.3, QUANTIFOIL). After the solvent was almost evaporated, the grid was plunged into liquid nitrogen rapidly. The grid was then transferred to the Fischone 2550 cryo holder and TEM at liquid nitrogen temperature. The cRED data were collected on a JEOL 2100-plus TEM equipped with MerlinEM direct electron detector under 200 kV acceleration voltage and installed with Heimdall data collection software (software developed by the ReadCrystal Tech Co.). The tilting range depends on the location of the crystals on the grid. Each frame was collected with exposure time of 1 s, resulting in a 1° wedge per frame. The data were visualized with program REDp (Wan et al., 2013) and processed using XDS (Kabsch, 2010) with the aid of with the aid of Coeus (software developed by ReadCrystal Tech Co.) for batch processing and merging.

## Results and Discussion

The sample for the CS method was prepared by the following steps: 1) synthesizing host, 2) exchanging solvent, and 3) soaking the molecule of interest. The pores of the as-made CS crystal were filled the nitrobenzene. Due to the high affinity between nitrobenzene and the host, the target molecule cannot be absorbed efficiently into the as-made sample. Thus, it is necessary to exchange the nitrobenzene to a more inert solvent, typically cyclohexane, to facilitate the inclusion of the target molecule. After solvent exchange, the target compound can be absorbed more efficiently into the host.

As a proof-of-concept experiment, we chose to elucidate the structure change in the three phases of the CS method: 1) as synthesized, 2) solvent exchanged, and 3) guest included. The crystalline sponge [(ZnI_2_)_3_-(tpt)_2_·x(Guest)]_n_ (noted as *1-Guest*) was synthesized following the protocol of Fujita (Hoshino et al., 2016), using nitrobenzene as the solvent. The as-made sample was donated as *1-Nitrobenzene*. Instead of the large crystals used in the traditional CS method, we are more interested about the powder-like crystals at the bottom of the synthesis liquid. Crystals in a size of few hundred nanometers are better for a guest molecule to defuse and also suitable for 3D-ED data collection. To exchange the solvent in the pore from nitrobenzene (high affinity to host) to cyclohexane (less affinity) for better guest-soaking result, the powder-like *1-Nitrobenzene* was washed with cyclohexane several times and soaked in it at 50°C for 2 h (donated as 1-Cyclohexane). The IR spectroscopy signal at 1,346 cm^−1^, assigned to nitrobenzene, completely disappeared (Supplementary Figure S1), indicating that the nitrobenzene within the pore was exchanged by cyclohexane completely. The exchange process made a great improvement in time versus a 7-day-exchange time in the original method using micron-sized crystal. We chose guaiazulene as our target molecule because it was widely tested from different groups and different light sources [Mo (Inokuma et al., 2013), Cu (Hoshino et al., 2016), and synchrotron (Ramadhar et al., 2015)]. The guest was included by slow-evaporation method. *1-Cyclohexane* was soaked in the cyclohexane solution of guaiazulene (1 mg/ml) in a pierced capped vial at 50°C for 12 h and then transferred to 4°C for storage. This guest-soaked sample was noted as *1-Guaiazulene*. The guest inclusion time (12 h) needed in this work was about fourfold faster than that in the original protocol (2 days) (Hoshino et al., 2016). In addition, these parameters could be further optimized to achieve faster guest exchange.

**FIGURE 1:**
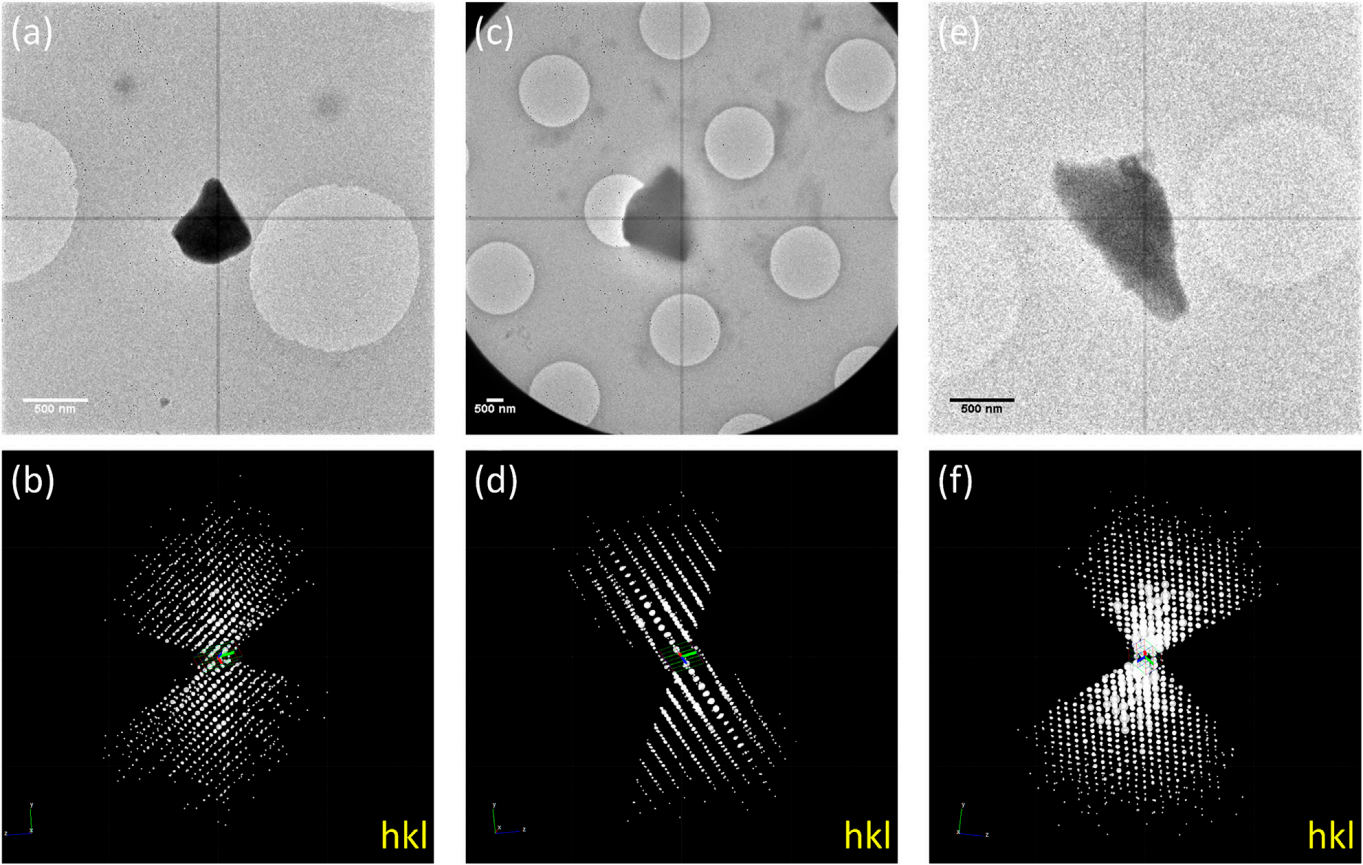
TEM images of **(A)** 1-Nitrobenzene, **(C)** 1-Cyclohexane, and **(E)** 1-Guaiazulene and the 3D reciprocal lattice of **(B)** 1-Nitrobenzene, **(D)** 1-Cyclohexane, and **(F)** 1-Guaiazulene.

**FIGURE 2:**
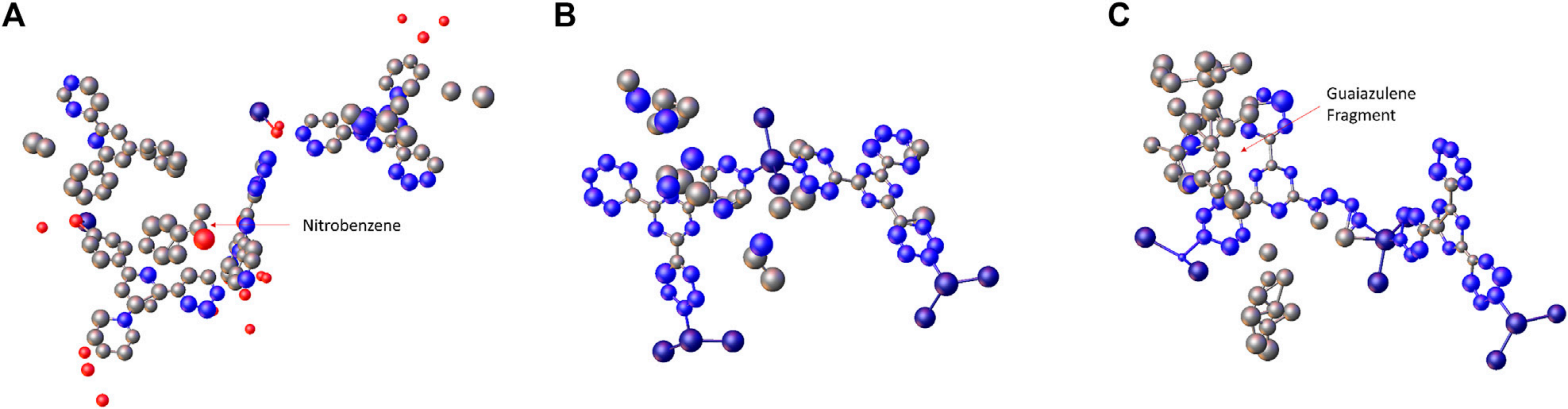
The initial structure model of **(A)** 1-Nitrobenzene, **(B)** 1-Cyclohexane, and **(C)** 1-Guaiazulene obtained from SHELXT.

The as-made (*1-Nitrobenzene*), solvent-exchanged (*1-Cyclohexane*), and guest-included (*1-Guaiazulene*) samples were transferred to TEM, and 3D-ED data under cryogenic temperature were collected to prevent beam damage and the removal/shifting of guest molecules. Based on our experience, the size of the crystal ranging from 500 nm to 1 μm is optimum for 3D-ED data collection. Crystals in this size are large enough to have strong diffractions, yet not too thick for electron to penetrate. The tilting range depends on the crystals’ location on the grid. Each frame was collected with an exposure time of 1 s, resulting in a 1° wedge per frame. To keep diffraction points well-separated, 40 cm of camera length was selected. Thus, the resolution was limited by the detector edge to 1 Å. However, some high-resolution points were still observable at the corner of the detector. The 3D-ED data were processed and merged by XDS (Kabsch, 2010). As shown in Figure 1, the reconstructed 3D reciprocal lattices suggested that *1-Nitrobenzene* crystallized in $P\bar{1}$ with unit cell of *a* = 13.810(3) Å, *b* = 16.550(3) Å, *c* = 26.130(5) Å, *α* = 89.87(3)°, *β* = 76.74(3)°, *γ* = 74.35(3)°; *1-Cyclohexane* crystallized in C2/c with unit cell of *a* = 34.560(7) Å, *b* = 14.520(3) Å, *c* = 30.860(6) Å, *α* = *γ* = 90°, *β* = 100.73(3)°; *1-Guaiazulene* crystallized in C2/c with unit cell of *a* = 33.490(7) Å, *b* = 14.180(3) Å, *c* = 29.740(6) Å, *α* = *γ* = 90°, *β* = 102.09(3)°. Since these crystals exhibit lower symmetry, merging multiple datasets is needed in order to increase the completeness. The reflection statistics of each merged dataset is listed in Table 1. *Ab initio* structure solution was performed by SHELXT (Sheldrick, 2015b) with intrinsic phasing methods. As shown in Figure 2, every non-H atom position of the framework in the three structures could be located directly, including guest molecules. Subsequent structure refinement was carried out by SHELXL (Sheldrick, 2015a). AFIX constraints were applied to the benzene and pyridyl groups, and soft restrains were applied to the guest molecules. The framework atoms were refined using an anisotropic atomic displacement factor with RIGU restraint. The guest molecules were refined using an isotropic atomic displacement factor for refinement stability.

**TABLE 1:** The crystallographic statistics and refinement detail of 1-Nitrobenzene, 1-Cyclohexane, and 1-Guaiazulene.

Identification code	1-Nitrobenzene	1-Cyclohexane	1-Guaiazulene
Empirical formula	C_37.95_H_26.05_I_6_N_12.91_O_0.82_Zn_3_	C_36_H_24_I_6_N_12_Zn_3_	C_43.21_H_32.65_I_6_N_12_Zn_3_
Formula weight	1,633.24	1,582.24	1,677.55
Temperature/K	100(2)	100(2)	100(2)
Crystal system	Triclinic	Monoclinic	Monoclinic
Space group	$P\bar{1}$	*C2/c*	*C2/c*
a/Å	13.810(3)	34.560(7)	33.490(7)
b/Å	16.550(3)	14.520(3)	14.180(3)
c/Å	26.130(5)	30.860(6)	29.740(6)
α/°	89.87(3)	90	90
β/°	76.74(3)	100.73(3)	102.09(3)
γ/°	74.35(3)	90	90
Volume/Å^3^	5,586(2)	15,215(5)	13,810(5)
Z, Z′	4, 2	8, 1	8, 1
ρ_calc_g/cm^3^	3.474	1.381	1.614
F(000)	1,382	1,686	1,861
Radiation/Å	Electron (*λ* = .02506)	Electron (*λ* = .02506)	Electron (*λ* = .02506)
Resolution cutoff/Å	1.0	1.0	1.0
2Θ range for data collection/°	.09–1.8	.084–1.436	.088–1.436
Index ranges	−16 ≤ h ≤ 16	−34 ≤ h ≤ 34	−33 ≤ h ≤ 33
	−20 ≤ k ≤ 20	−14 ≤ k ≤ 14	−14 ≤ k ≤ 13
	−30 ≤ l ≤ 32	−30 ≤ l ≤ 30	−29 ≤ l ≤ 29
Reflections collected	88,206	44,654	96,164
Completeness/%	99.2	99.6	99.8
Independent reflections	11,608	7,954	7,215
	*R*_int_ = .2497	*R*_int_ = .3090	*R*_int_ = .5389
	*R*_sigma_ = .1557	*R*_sigma_ = .2167	*R*_sigma_ = .2162
Data/restraints/parameters	11,608/936/908	7,954/126/418	7,215/825/474
Goodness-of-fit on F^2^	1.759	1.298	1.502
Final R indexes [I> = 2σ (I)]	*R*_1_ = .2265, w*R*_2_ = .5205	*R*_1_ = .2192, w*R*_2_ = .4815	*R*_1_ = .2276, w*R*_2_ = .5211
Final R indexes [all data]	*R*_1_ = .2664, w*R*_2_ = .5397	*R*_1_ = .3149, w*R*_2_ = .5422	*R*_1_ = .3007, w*R*_2_ = .5524
Largest diff. peak/hole/e Å^−3^	.54/−.37	.29/−.30	.46/−.27

**FIGURE 3:**
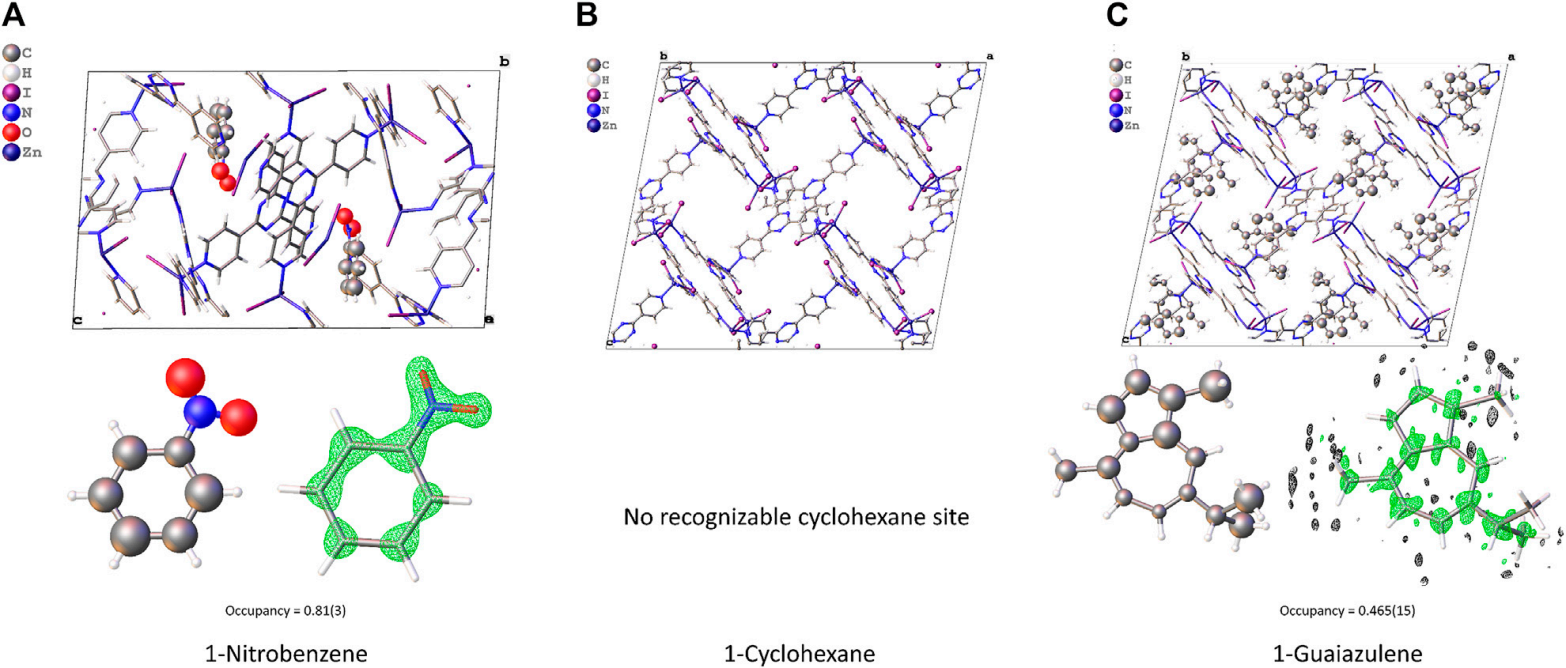
The structure model, guest molecule conformation, and F_o_ density map (drawn in 0.5e-/Å^3^ level) of **(A)** 1-Nitrobenzene, **(B)** 1-Cyclohexane, and **(C)** 1-Guaiazulene.

**FIGURE 4:**
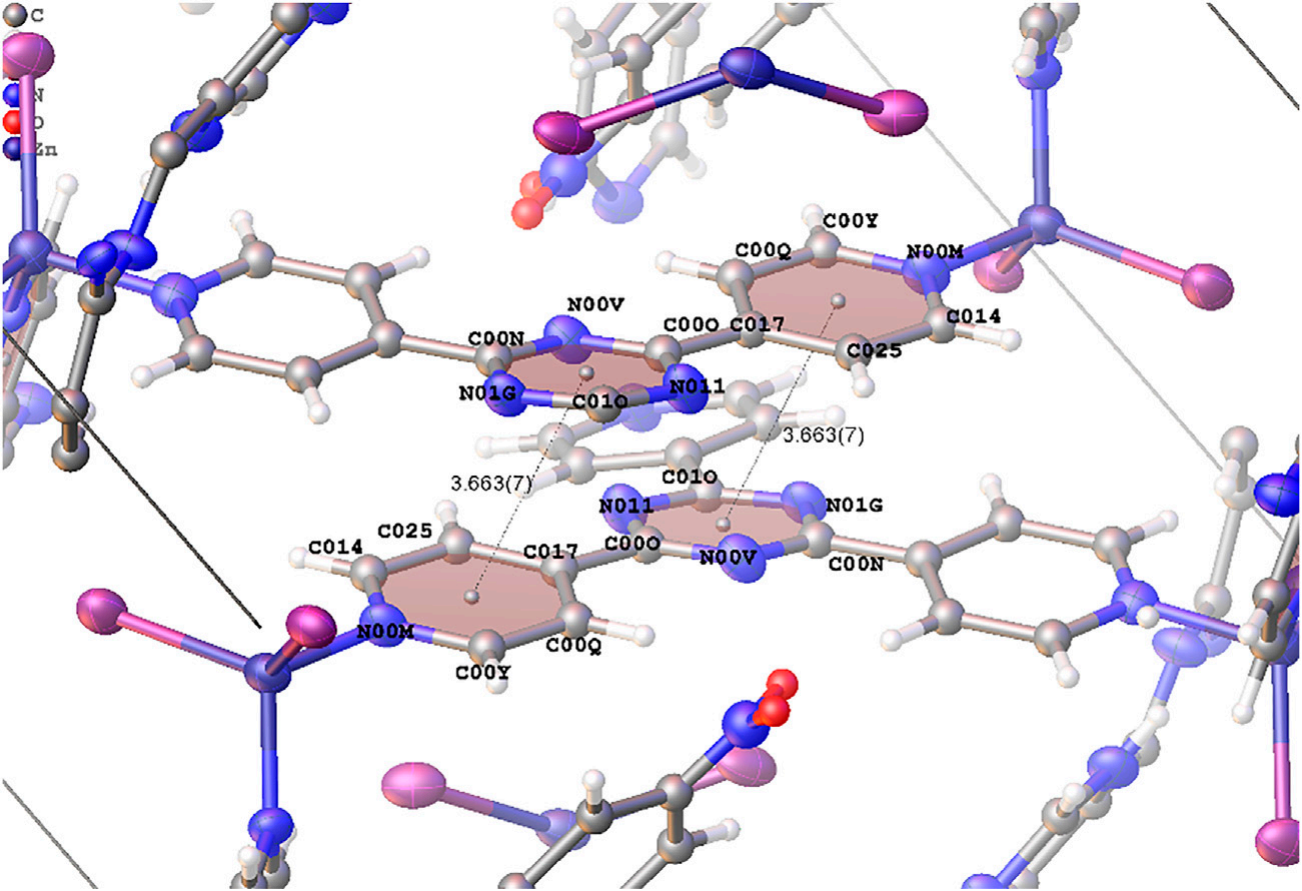
π–π interaction between pyridyl and triazine ring in different complex chains.

The final *R_1_* value converged to 0.2265, 0.2192, and 0.2276 for *1-Nitrobenzene*, *1-Cyclohexane*, and *1-Guaiazulene*, respectively. The refined model is shown in Figure 3. The pore size and cell volume of *1-Nitrobenzene* are much smaller than the other samples due to the π–π interaction between two complex chains. As shown in Figure 4, the two ligands are offset stacked with their centroid distance of 3.663(7) Å. One nitrobenzene molecule is found in *1-Nitrobenzene*, with its occupancy of 0.81(3). After solvent exchange, the framework is considerably expanded. No obvious residue electron density peaks in *1-Cyclohexane* could assign to cyclohexane, and the *R_1_* value is comparable with the others. Thus, further SQUEEZE (Spek, 2015) procedure is not needed. For *1-Guaiazulene*, the missing part of the target molecule could be found on the difference Fourier map after few rounds of refinement. The low occupancy of guaiazulene, 0.465(15), leads to a slightly inaccurate bond length/angle and slightly distorted conformation. It is worth noting that these three samples have a large fraction of empty void where the residue peaks could not assign to any solvent molecules. The SCXRD CS method often suffers from disordered solvent molecules. It blurs the electron density distribution and complicates the structure refinement. However, in this case, the high-vacuum environment in the TEM chamber may extract the solvent molecules that have a weaker interaction with the host. Without the interference of a disordered solvent, target molecules with low occupancy can still be recognized.

**FIGURE 5:**
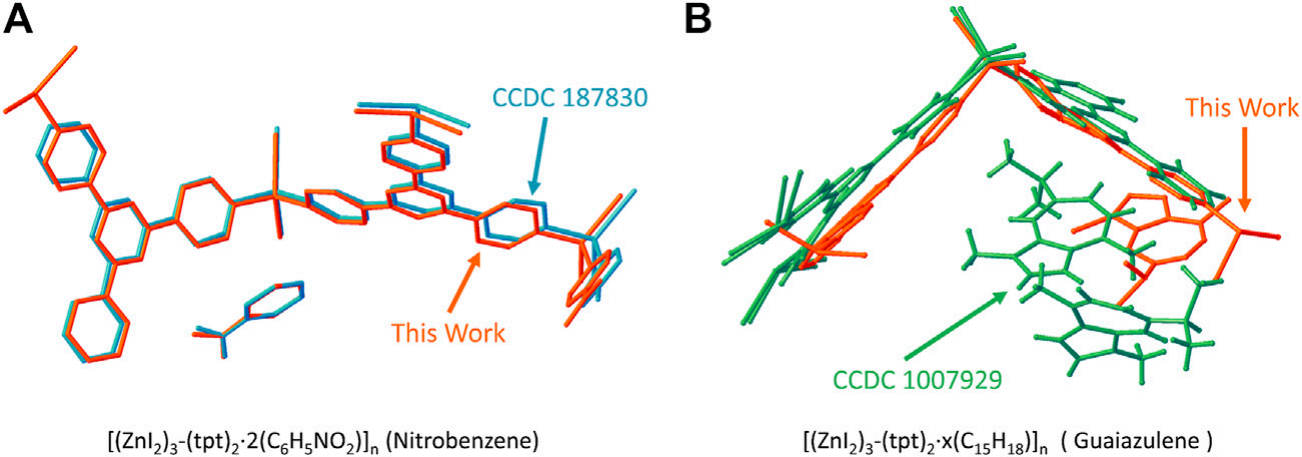
The comparison of the structure model in this work and the reported one for **(A)** [(ZnI_2_)_3_-(tpt)_2_·2(C_6_H_5_NO_2_)]_n_ (1-Nitrobenzene) and CCDC 187830 (Biradha and Fujita, 2002) and **(B)** [(ZnI_2_)_3_-(tpt)_2_·x(C_15_H_18_)]_n_ (1-Guaiazulene) and CCDC 1007929 (Ramadhar et al., 2015).

If we compare the structure solved by 3D-ED with respect to the reported ones, as shown in Figure 5, the model for *1-Nitrobenzene* solved by 3D-ED and that by x-ray (CCDC 187830) (Biradha and Fujita, 2002) are almost identical. For *1-Guaiazulene*, the framework position is slightly distorted and the guest molecule location and number were totally different to the synchrotron-resolved one (Ramadhar et al., 2015). We believe this difference originated from the guest-soaking process. The [(ZnI_2_)_3_-(tpt)_2_]_n_ framework is flexible to the response of the guest. Therefore, the guaiazulene-soaking condition will influence the fine structure, i.e., host conformation, number, and the position of the guests, dramatically. The guest location and occupancy in the literature-reported ones are also different (Inokuma et al., 2013; Ramadhar et al., 2015; Hoshino et al., 2016). This result also demonstrates the accuracy of the 3D-ED in the structure determination routine.

## Conclusion

In conclusion, the crystalline sponge method was successfully carried out by the 3D-ED technique using [(ZnI_2_)_3_-(tpt)_2_·x(Guest)]_n_ porous complex nanocrystals. Utilizing the better diffuse ability of small crystals, the time needed for solvent-exchange and guest-soaking protocols were shortened to 2 and 12 h respectively, about 50-fold faster versus the original protocol. It should be noticed that these conditions can be further optimized. The structures of *1-Nitrobenzene, 1-Cyclohexane, and 1-Guaiazulene* were fully resolved using a merged dataset. The structure model was identical to previously reported ones using x-ray, showing that the accuracy of the 3D-ED was comparable with SCXRD. The occupancy of the guest molecule, nitrobenzene and guaiazulene, were 0.81(3) and 0.465(15), respectively. This work not only provides a new data collection strategy for crystalline sponge method but also demonstrates the potential of the 3D-ED techniques to study host–guest interaction by solving fine structures of porous materials.

## Data Availability

The datasets presented in this study can be found in online repositories. The names of the repository/repositories and accession number(s) can be found in the article/Supplementary Material. The final structures (coordinates, reflections, and structure factors) of 1-Nitrobenzene, 1-Cyclohexane, and 1-Guaiazulene have been deposited in the Cambridge Crystallographic Data Center (CCDC). The CCDC numbers of the samples are 2124118, 2124119, 2124120.
