# Supplementary material for: Crystalline Sponge Method by Three-Dimensional Electron Diffraction
Source: Front Mol Biosci. 2022 Feb 7;8:821927. doi: 10.3389/fmolb.2021.821927 (PMC8859408; doi:10.3389/fmolb.2021.821927)
Supplement: Supplementary file 2 [file DataSheet1.DOCX]

Supplementary Material

# Supplementary Figures and Tables


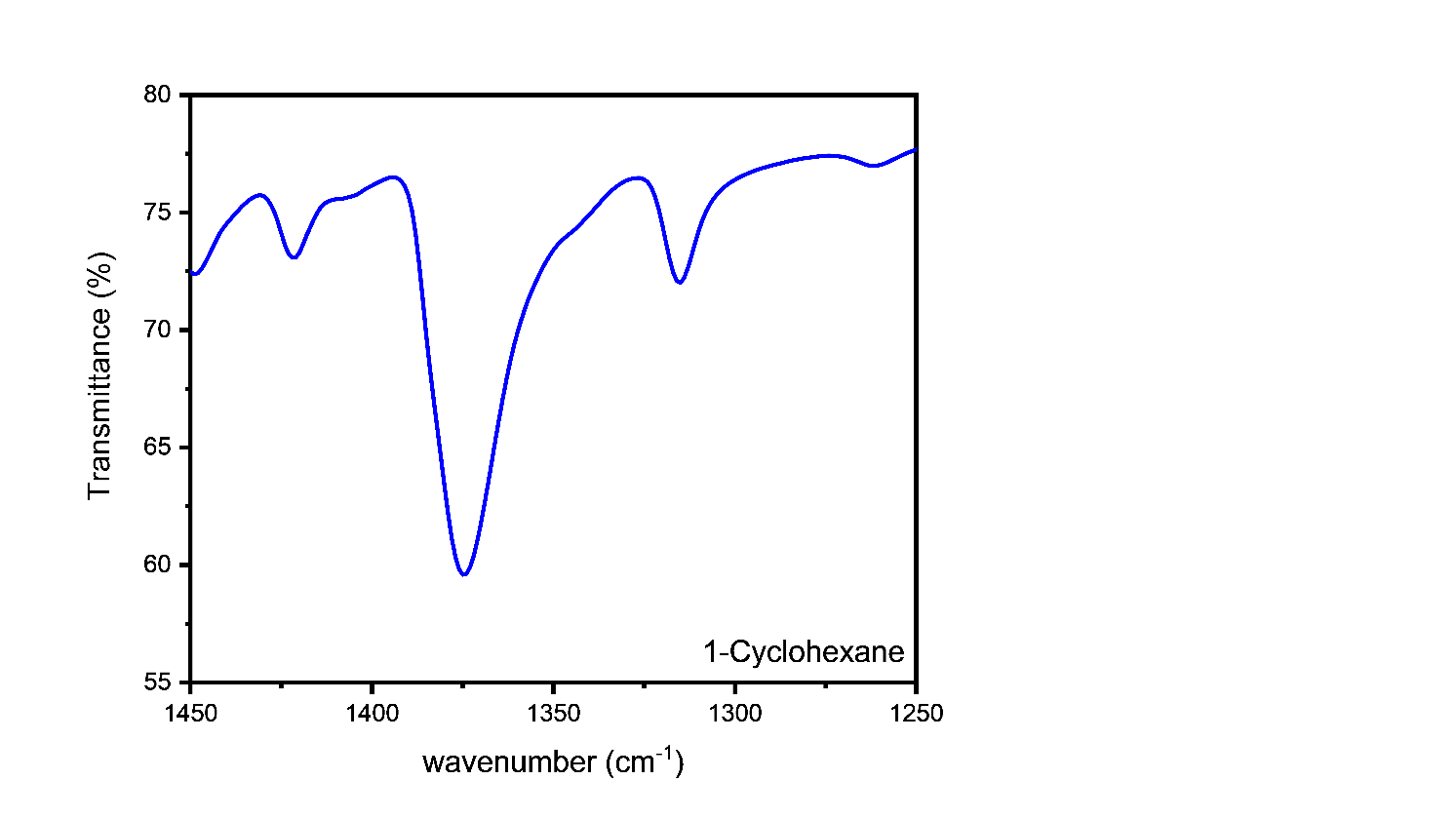


Figure S1 The IR spectrum of 1-cyclohexane. No peaks observed at 1346cm^-1^ indicated the complete exchange of nitrobenzene.

Table S1 The reflection statistics of 12 individual datasets of 1-Nitrobenzene

| # | *a/Å* | *b/Å* | *c/Å* | *α/°* | *β/°* | *γ/°* | Volume/ *Å^3^* | Rotation angle*°* | n_tot_ | n_uniq_ | Completeness/% | I/σ | r_meas_ | CC_1/2_ |
| --- | --- | --- | --- | --- | --- | --- | --- | --- | --- | --- | --- | --- | --- | --- |
| 1 | 14.10 | 16.7 | 26.72 | 89.71 | 104.13 | 105.64 | 5863.23 | 81.56 | 12162 | 7864 | 35.1 | 1.79 | 22.3 | 98.9 |
| 2 | 13.97 | 17.01 | 26.42 | 89.24 | 103.58 | 105.78 | 5864.21 | 73.05 | 11095 | 7308 | 32.7 | 3.52 | 11.4 | 99.6 |
| 3 | 14.12 | 16.75 | 26.68 | 90.48 | 103.52 | 104.32 | 5929.72 | 81.34 | 9617 | 6163 | 27.9 | 2.95 | 18.8 | 97.5 |
| 4 | 13.62 | 16.52 | 26.05 | 90.35 | 102.76 | 104.70 | 5517.35 | 123.05 | 10583 | 6684 | 30.0 | 3.07 | 16.3 | 99.3 |
| 5 | 13.7 | 16.46 | 25.99 | 90.50 | 103.35 | 104.84 | 5497.90 | 111.55 | 11603 | 7275 | 32.8 | 1.58 | 29.7 | 97.7 |
| 6 | 13.86 | 16.37 | 25.98 | 89.42 | 103.17 | 106.33 | 5498.83 | 95.84 | 8705 | 5436 | 24.4 | 3.06 | 15.1 | 99.3 |
| 7 | 13.72 | 16.64 | 25.95 | 90.45 | 103.10 | 105.71 | 5539.46 | 105.87 | 9415 | 5910 | 26.8 | 3.4 | 13.7 | 99.3 |
| 8 | 13.67 | 16.59 | 25.94 | 89.38 | 102.67 | 106.01 | 5509.35 | 116.45 | 11496 | 7012 | 32.2 | 3.67 | 14.7 | 98.9 |
| 9 | 13.84 | 16.44 | 26.03 | 90.23 | 103.10 | 106.04 | 5530.08 | 87.26 | 9967 | 5928 | 27.0 | 3.62 | 16.6 | 98.9 |
| 10 | 13.83 | 16.33 | 25.91 | 89.96 | 102.97 | 105.89 | 5472.90 | 94.20 | 16161 | 10096 | 42.2 | 2.62 | 20.3 | 98.1 |
| 11 | 13.64 | 16.57 | 26.18 | 88.88 | 102.80 | 105.91 | 5543.70 | 117.65 | 14598 | 9060 | 37.8 | 2.23 | 27.1 | 98.1 |
| 12 | 13.86 | 16.46 | 26.03 | 89.98 | 103.67 | 105.71 | 5541.70 | 100.10 | 15352 | 9965 | 41.3 | 1.02 | 62.9 | 94.0 |

Table S2 The reflection statistics of 4 individual datasets of 1-Cyclohexane

| # | *a/Å* | *b/Å* | *c/Å* | *α/°* | *β/°* | *γ/°* | Volume/ *Å^3^* | Rotation angle*°* | n_tot_ | n_uniq_ | Completeness/% | I/σ | r_meas_ | CC_1/2_ |
| --- | --- | --- | --- | --- | --- | --- | --- | --- | --- | --- | --- | --- | --- | --- |
| 1 | 34.94 | 14.65 | 31.11 | 90.00 | 102.10 | 90.00 | 15570.35 | 108.0474 | 27613 | 14530 | 87.4 | 1.02 | 32.3 | 98.5 |
| 2 | 35.58 | 14.65 | 31.09 | 90.00 | 101.23 | 90.00 | 15895.12 | 84.2595 | 21775 | 11846 | 68.7 | 0.78 | 47.2 | 98.5 |
| 3 | 33.87 | 14.49 | 30.75 | 90.00 | 102.05 | 90.00 | 14759.06 | 100.8246 | 13360 | 7005 | 45.0 | 1.51 | 32.5 | 98 |
| 4 | 33.93 | 14.35 | 30.57 | 90.00 | 99.93 | 90.00 | 14661.50 | 66.586 | 8650 | 4317 | 28.2 | 2.43 | 22.9 | 99.1 |

Table S3 The reflection statistics of 12 individual datasets of 1-Guaiazulene

| # | *a/Å* | *b/Å* | *c/Å* | *α/°* | *β/°* | *γ/°* | Volume/ *Å^3^* | Rotation angle*°* | n_tot_ | n_uniq_ | Completeness/% | I/σ | r_meas_ | CC_1/2_ |
| --- | --- | --- | --- | --- | --- | --- | --- | --- | --- | --- | --- | --- | --- | --- |
| 1 | 33.53 | 14.22 | 30.11 | 90 | 101.37 | 90 | 14074.60 | 78.74 | 20762 | 13871 | 46.7 | 1.13 | 31.4 | 98.8 |
| 2 | 33.92 | 14.33 | 29.82 | 90 | 101.35 | 90 | 14211.05 | 77.70 | 10329 | 4718 | 31.2 | 2.27 | 28.5 | 98.0 |
| 3 | 33.48 | 14.29 | 30.16 | 90 | 101.66 | 90 | 14131.61 | 90.69 | 12218 | 6263 | 42.1 | 1.82 | 31.6 | 98.3 |
| 4 | 33.94 | 14.27 | 29.82 | 90 | 102.97 | 90 | 14074.13 | 85.18 | 11272 | 6733 | 45.3 | 1.73 | 30.4 | 98.1 |
| 5 | 33.83 | 14.16 | 29.73 | 90 | 101.76 | 90 | 13942.51 | 89.92 | 11915 | 6150 | 41.8 | 1.9 | 30.9 | 98.5 |
| 6 | 33.77 | 14.18 | 29.87 | 90 | 101.12 | 90 | 14034.92 | 91.02 | 11856 | 6155 | 41.5 | 2.45 | 24.5 | 99.1 |
| 7 | 33.45 | 14.32 | 29.83 | 90 | 103.03 | 90 | 13921.01 | 98.60 | 13065 | 7128 | 47.6 | 1.36 | 33.4 | 95.4 |
| 8 | 32.95 | 14.19 | 29.23 | 90 | 101.82 | 90 | 13377.20 | 75.29 | 9199 | 4317 | 30.6 | 1.13 | 32.4 | 95.3 |
| 9 | 33.04 | 14.02 | 29.46 | 90 | 102.27 | 90 | 13334.96 | 80.33 | 9982 | 5298 | 37.0 | 1.29 | 19.4 | 98.7 |
| 10 | 33.35 | 13.99 | 29.55 | 90 | 102.94 | 90 | 13437.19 | 78.56 | 9609 | 4931 | 34.7 | 0.73 | 28.3 | 97.5 |
| 11 | 33.11 | 14.04 | 29.56 | 90 | 102.76 | 90 | 13402.08 | 80.50 | 9598 | 5658 | 39.8 | 1.11 | 40.7 | 96.1 |
